# The visualization of hypertrophic pachymeningitis in antineutrophil cytoplasmic antibody-associated granulomatosis with polyangiitis on contrast-enhanced FLAIR

**DOI:** 10.1016/j.radcr.2023.10.041

**Published:** 2023-11-14

**Authors:** Satoshi Yoshikawa, Takeshi Ueda

**Affiliations:** Department of Emergency and General Internal Medicine, Rakuwakai Marutamachi Hospital, 9-7 Jurakumawari-Matsushita-cho, Nakagyo-ku, Kyoto 604-8401, Japan

**Keywords:** Hypertrophic pachymeningitis, Antineutrophil cytoplasmic antibody-associated vasculitis, Contrast-enhanced FLAIR

## Abstract

Hypertrophic pachymeningitis is a rare inflammatory condition that leads to the thickening of the dura mater, either due to unknown or identifiable secondary causes. Granulomatosis with polyangiitis is a notable causative agent, and hypertrophic pachymeningitis is the initial presentation in certain cases. The diagnosis of hypertrophic pachymeningitis is aided by contrast-enhanced MRI, although distinguishing between normal and abnormal dural enhancement can be challenging using contrast-enhanced T1WI. This study highlights the case of an 80-year-old woman diagnosed with hypertrophic pachymeningitis secondary to antineutrophil cytoplasmic antibody-associated granulomatosis with polyangiitis, where contrast-enhanced FLAIR played a crucial role in distinctly identifying abnormal dural enhancement and differentiating it from normal dura. In conclusion, although contrast-enhanced T1WI remains indispensable, contrast-enhanced FLAIR can serve as a valuable complementary tool in MRI study sequences for the diagnosis of hypertrophic pachymeningitis.

## Introduction

Hypertrophic pachymeningitis (HP) is a rare form of diffuse inflammatory disease that causes thickening of the dura mater. It can involve the cranial or the spinal dura or both [1]. HP can be divided into 2 forms: primary or idiopathic hypertrophic pachymeningitis, where no identifiable cause is found, and secondary hypertrophic pachymeningitis, where identifiable causes coexist, such as neoplasm, trauma, infections, or autoimmune diseases. Within the scope of immune-mediated diseases, HP can appear as a manifestation of immunoglobulin G4-related disease, neurosarcoidosis, granulomatosis with polyangiitis (GPA) [2], microscopic polyangiitis [3], polyarteritis nodosa [4], rheumatoid arthritis [5], and Sjögren syndrome [6]. GPA is a subtype of antineutrophil cytoplasmic antibody (ANCA)-associated vasculitis (AAV). It can appear in a specific area or present as a disease affecting multiple organs [7]. In 7%-11% of patients with GPA, central nervous system (CNS) symptoms are seen, typically presenting in 3 main ways: affecting the blood vessels in the brain, involving the pituitary gland, and impacting the leptomeninges [8–10]. Although other types of AAV (eg, microscopic polyangiitis and eosinophilic granulomatosis with polyangiitis) can cause HP, patients with HP are most frequently classified as GPA [11]. In an analysis of a study discussing CNS symptoms in GPA patients, 60% of the HP cases linked to GPA showed HP as their first symptom [9]. Contrast-enhanced MRI (CE-MRI) plays an important role in diagnosing HP because of the high sensitivity [11], although normal dura can be occasionally depicted on contrast-enhanced T1WI (CE-T1WI) [12]. Contrast-enhanced FLAIR (CE-FLAIR) was recently reported to be useful for detecting the abnormalities in the leptomenengeal space [13]. However, to the best of our knowledge, no reports have discussed the utility of CE-FLAIR for pachymeningeal abnormalities of AAV. Herein, we report the usefulness of CE-FLAIR for the detection of pachymeningitis associated with AAV.

## Case

An 80-year-old woman presented with a fever of 38.0°C and altered visual acuity for 1 week. She reported no headache, arthralgia, numbness, cranial nerve paralysis, hematuria, or rashes. Laboratory findings indicated leukocytosis, and a C-reactive protein level of 20.88 mg/dL. Serological tests for syphilis, antihuman immunodeficiency virus antibodies, hepatitis B surface antigen, antihepatitis C virus antibodies, antinuclear antibodies, and anti-Ro/SSA antibodies were negative. Both serum Angiotensin-converting enzyme and IgG4 levels were within normal limits. Acid-Fast Bacilli culture of the cerebrospinal fluid was also negative. Ophthalmological assessment showed light perception in her right eye with a pressure of 23.0 mm Hg, while her left visual acuity was noted to be a numerus digitorum. Further examination revealed left dominant bilateral optic neuritis and posterior uveitis. The otorhinolaryngological findings were unremarkable, with no hearing loss or normal tympanic membranes. CE-MRI was conducted, suspecting ANCA-associated vasculitis, despite both myeloperoxidase-ANCA and proteinase-3-ANCA testing being negative.

**Fig. 1:**
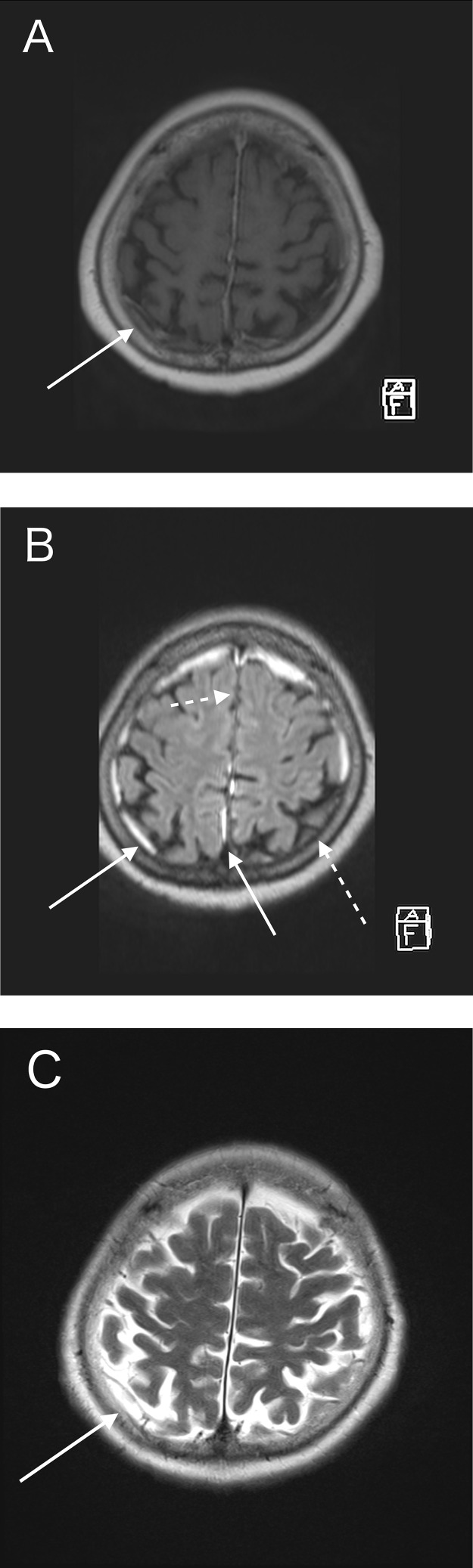
(A) CE-T1WI showed enhancement of the convexity of the dura and falx (arrow). (B) CE-FLAIR prominently visualized these lesions (arrows) against the backdrop of nonenhanced normal dura (dashed arrows). (C) T2WI demonstrated high intensity in the thickened convexity dura mater (arrow).

**Fig. 2:**
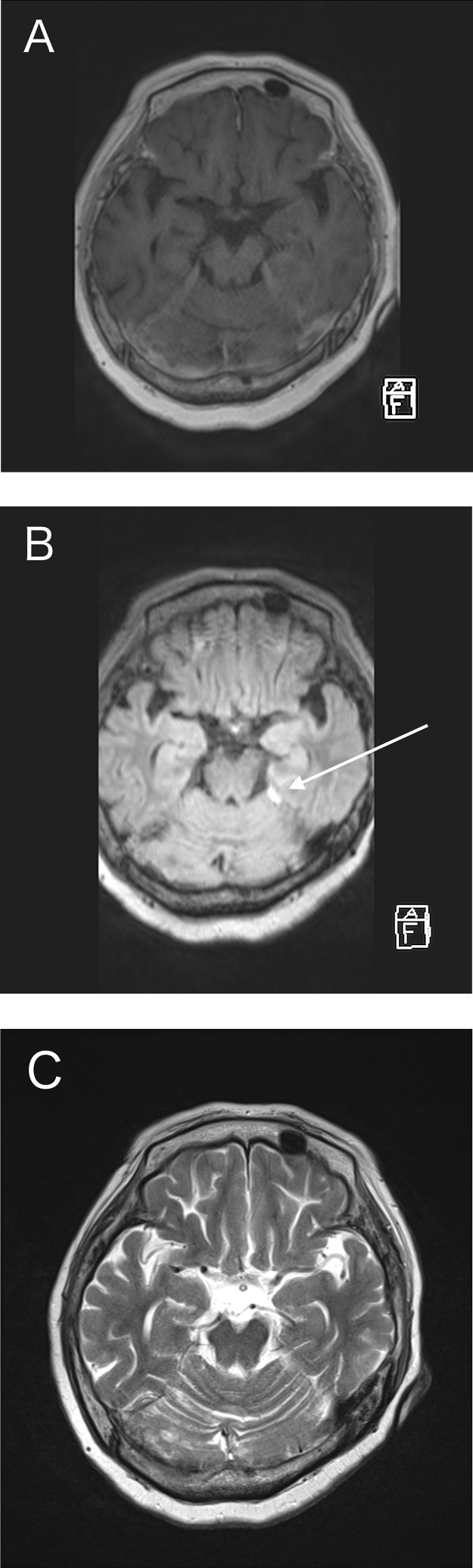
(A) CE-T1WI could not depict a thickened tentorium. (B) CE-FLAIR virtually demonstrated the thickened tentorium on the left side (arrow). (C) The tentorium was not detected on T2WI.

**Fig. 3:**
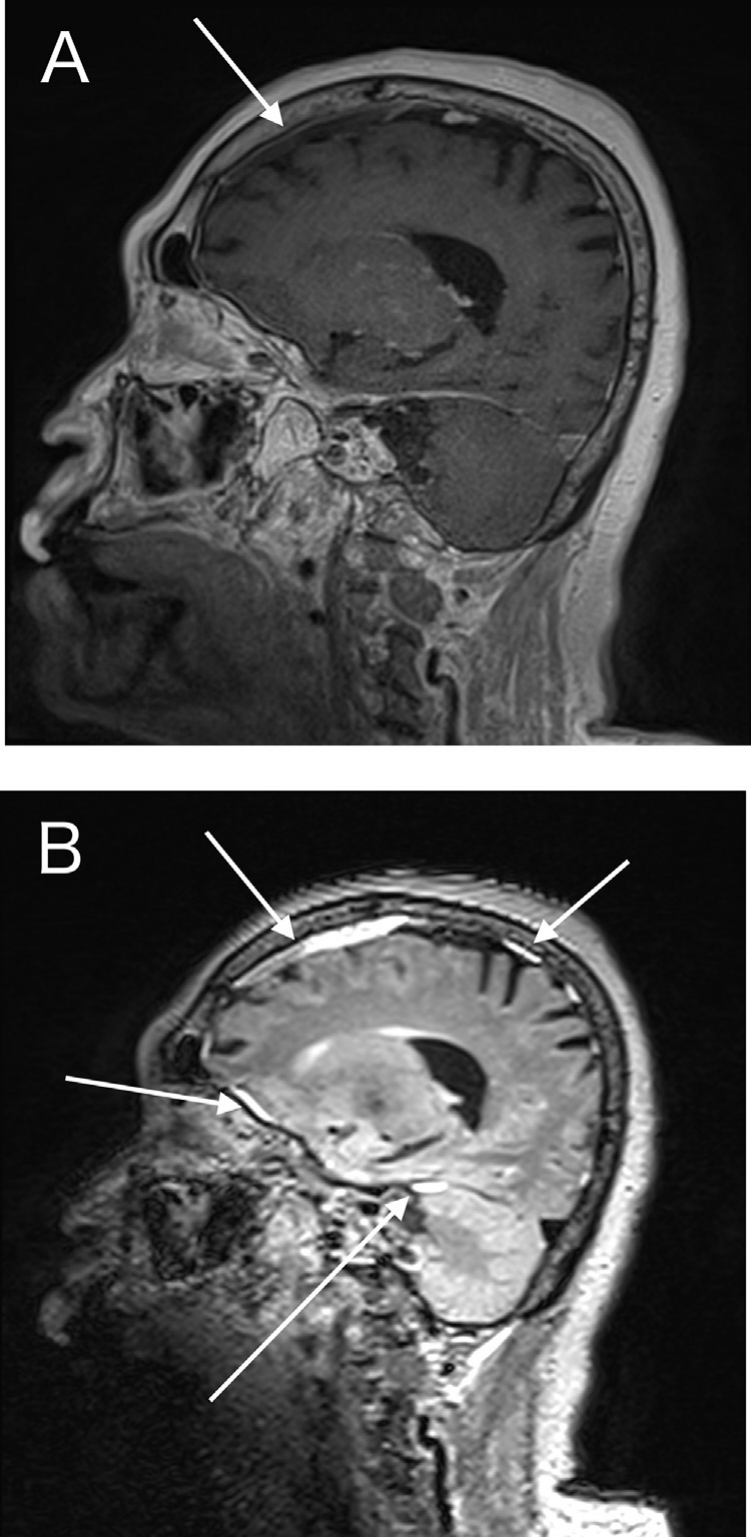
(A) CE-T1WI demonstrated a peripheral linear enhancement of the thickened falx (arrow). (B) The thickened falx was peripherally and partially homogeneously enhanced on the CE-FLAIR images (arrows). The homogeneous enhancement of the falx may reflect high sensitivity to low Gd concentrations in CE-FLAIR.

**Fig. 4:**
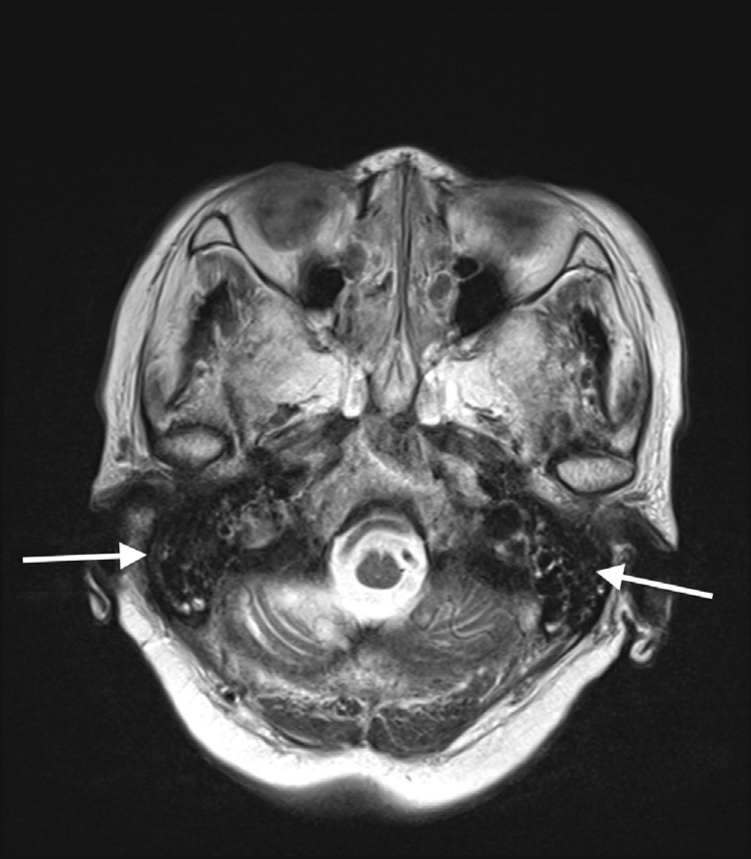
Fluid collections in the bilateral mastoid cells were noted on T2WI (arrows).

**Fig. 5:**
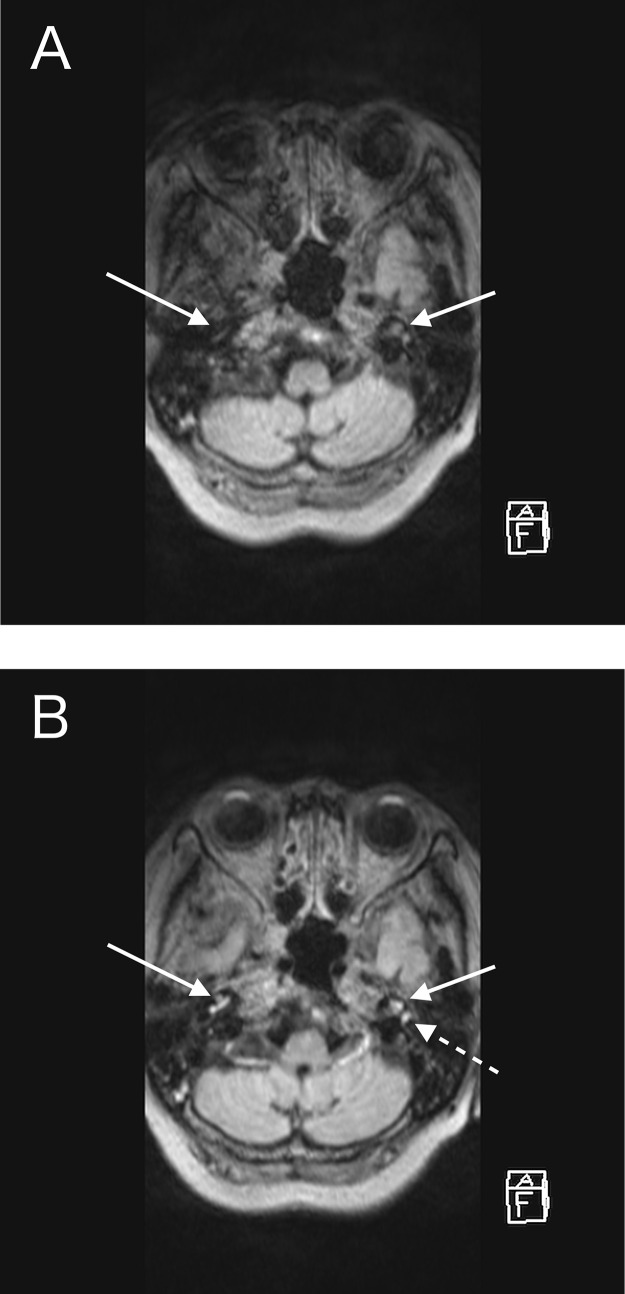
(A) Nonenhanced FLAIR showed isointensity of the chochleae and semicircular canals (arrows). (B) CE-FLAIR images showed bilateral enhancement of the cochlea (arrows) and semicircular canals (dashed arrow).

**Fig. 6:**
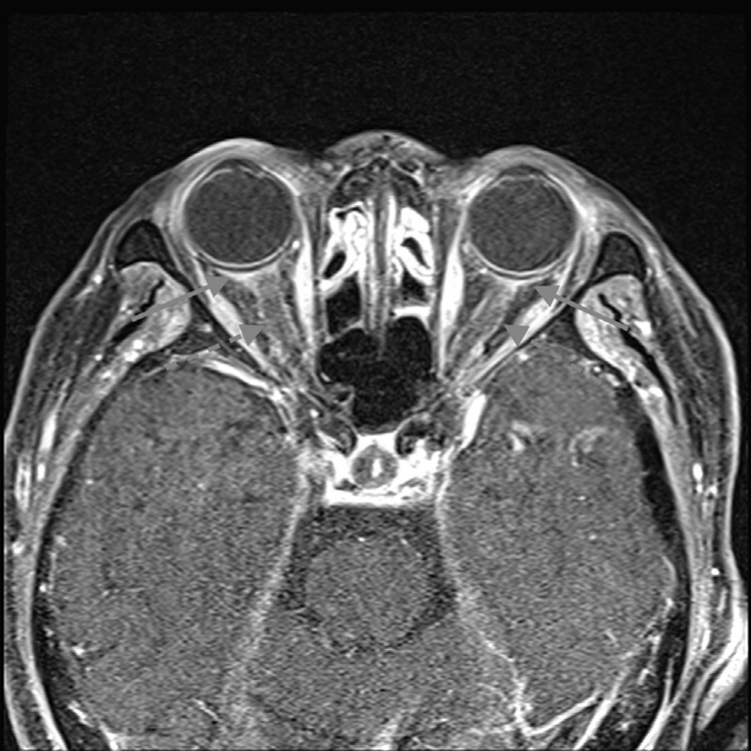
Fat-suppression orbital CE-T1WI revealed abnormal enhancement in the bilateral posterior eye globes (arrows) and optic nerves (dashed arrows), suggesting uveitis and optic neuritis.

CE-T1WI showed a thickened dura enhancement, making it challenging to differentiate it from usual dural enhancement (Fig. 1A). CE-FLAIR distinctly highlighted abnormal dural enhancement, without normal dural enhancement (Fig. 1B). This was particularly pronounced in the falx and the tentorium. CE-FLAIR prominently visualized the falx lesion against the backdrop of the non-enhanced normal falx (Figs. 2B and 3B). The thickened dura showed high intensity on T2WI (Fig. 1C), which implied an elevated free water content compared with the normal dura. T2WI revealed fluid accumulation in the mastoid cells, consistent with mastoiditis (Fig. 4) and CE-FLAIR also enhanced the cochleae and semicircular canals bilaterally (Figs. 5A and B), although the patient did not present with any hearing impairment. CE-T1WI showed enhancement in the posterior eye globes and optic nerves (Fig. 6), suggestive of posterior uveitis and optic neuritis. Based on these findings, hypertrophic pachymeningitis secondary to ANCA-associated granulomatosis with polyangiitis was diagnosed.

**Fig. 7:**
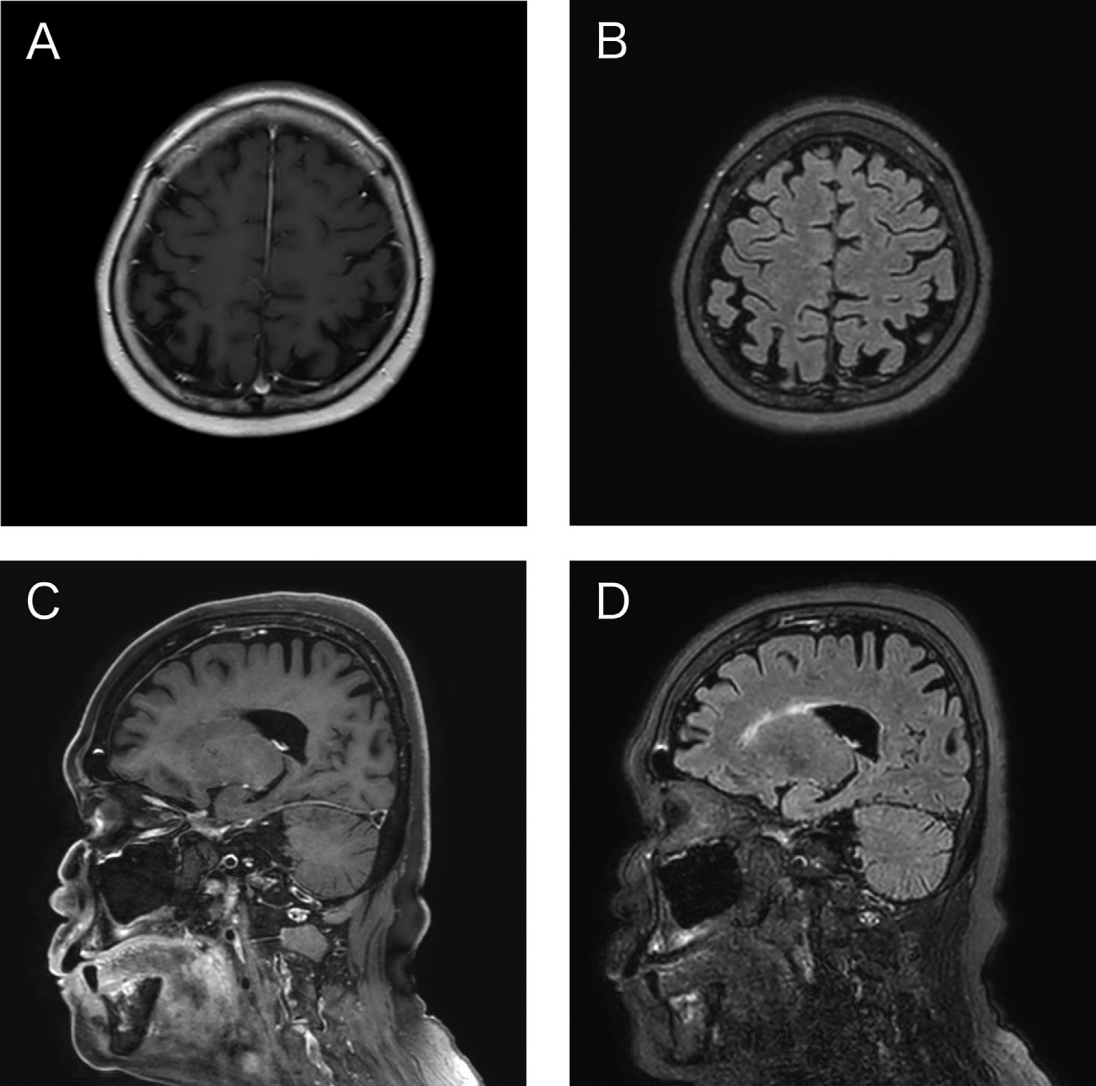
(A, C) CE-T1WI (B, D) CE-FLAIR CE-MRI performed 6 months post-treatment showed marked improvement in the thickened dural enhancement. While CE-T1WI demonstrated normal dural enhancement, CE-FLAIR revealed complete absence of enhanced dural matter.

The patient was treated with 1000 mg of intravenous methylprednisolone for 3 days and rituximab (500 mg). Despite this intervention, there was minimal improvement in her visual symptoms. CE-MRI performed 6 months post-treatment showed marked improvement in the thickened dural enhancement (Fig. 7), reduced fluid in the mastoid cells, and decreased enhancement in the eye globe and optic nerve. Notably, CE-FLAIR revealed a complete absence of enhanced dural matter, suggesting that the normal dural tissue was not visualized using CE-FLAIR.

## Discussion

The dura adjacent to the brain can be divided into 2 layers: the outer endosteal layer and the inner meningeal layer, neither of which has a BBB [12]. In most anatomical regions, the 2 dural layers remain conjoined; however, in specific areas, they diverge to constitute the dural venous sinuses. Furthermore, local infoldings of the meningeal layer of the dura constitute structures, such as the falx cerebri, tentorium cerebelli, and falx cerebelli. The dura mater consists of dense fibrous connective tissue which is tight enough not to be enhanced on CE-T1WI even without BBB because there is scarcely any extracellular space, and little free water that would undergo T1 shortening effects due to the contrast agent [12]. Nevertheless, the dura shows mild linear enhancement on CE-T1WI because many meningeal vessels in the dura are enhanced. In pathological processes such as inflammatory and neoplastic processes, an increase in the extracellular space, leading to an increase in the amount of both free water and contrast agent in the dura, strengthens the enhancement; therefore, it is theoretically distinguishable from normal dura matter. Nonetheless, it is sometimes difficult to differentiate abnormal dural enhancement from normal enhancement on CE-T1WI, particularly in the falx cerebri and tentorium cerebelli. It is not only because half of those parts of the dura can be enhanced even in the case of nonpathological process [12], but also because the abnormal dura is occasionally not sufficiently enhanced to be recognized, as in our case.

However, on CE-FLAIR, abnormal dural enhancement can be easily differentiated from normal dura, as observed in our case, for the following 2 reasons:

First, the pathological process affecting the dura is likely to manifest as heightened signal intensities in CE-FLAIR images. In an in vitro study, CE-FLAIR demonstrated superior sensitivity to low gadolinium (Gd) concentrations (0.01 mmol/L-0.5 mmol/L) compared to CE-T1WI [14]. This heightened sensitivity allows for a more distinct representation of tissues, especially those with low Gd concentrations such as the dura.

Second, the normal dura was scarcely visible on CE-FLAIR images. This might be attributed to the flow velocity reducing the enhancement of vessels. In the in vitro study, the signal intensity of the blood-mimicking fluids began to decrease as the blood flow velocity surpassed 1.0 mm/s on CE-FLAIR. In contrast, the signal intensity remained consistent on CE-T1WI regardless of the flow velocity. Although, to the best of our knowledge, no studies have directly examined dural flow velocity, the velocity of red blood cells (RBCs) within the cerebral capillaries has been reported to range between 0.3 and 3.2 mm/s [15]. Furthermore, the velocity is augmented in other vascular structures, such as arterioles and venules within the dura, and typically, the normal dura mater exhibits minimal enhancement on CE-FLAIR.

In conclusion, it can be postulated that pathological dura mater is distinctly delineated on CE-FLAIR, attributed to the pronounced intensity of the abnormal dura set against the subdued intensity of the normal dura. Nonetheless, given that CE-FLAIR has reduced sensitivity to high Gd concentrations (0.5 mmol/L-3.0 mmol/L) in comparison to CE-T1WI, it should not supplant CE-T1WI. Instead, CE-FLAIR should be incorporated as an adjunct to routine MRI sequences.

## Patient consent

Written informed consent for the publication of this case report was obtained from the patient.
